# A Case of Poisoning by Hyoscyamin Treated by Morphia Hypodermically

**Published:** 1885-11-20

**Authors:** Robert Coltman

**Affiliations:** Jenkintown, Pa.


### A CASE OF POISONING BY HYOSCYAMIN TREATED BY MORPHIA HYPODERMACALLY.

By Robert Coltman, M.D., of Jenkintown, Pa.

On the evening of June 27, 1885, about 7 o'clock, a gentleman of h'gh culture, and well versed in chemistry, pharmacy, also in general science, came to my office in a high state of nervous excitement, and told me he had taken about 1-60 of grain of hyoscyamin by the hypodermic syringe, a half hour previous to his coming to see me, and just before he came he had taken a grain of morphia by the mouth. Upon examination, I found the pupils dilated to their fullest extent ; pulse 155 and very feeble ; respiration rapid and shallow ; conjunctiva glazed and dry ; articulation impaired and the patient unable to walk without staggering and almost falling. His mind wandered, and he showed a great disposition to sleep. Upon being aroused from his naps, it took him a few minutes to recover his thoughts. He had been addicted to the morphia habit for several years, and had but recently succeeded in giving up the drug.

I immediately gave him ¼ of a grain of morphia hypodermacally, which affected the pulse and respiration to a perceptible extent in three minutes. As I kept him on my varanda in order to have plenty of fresh air, I could not see my watch to record the pulse, but found that both velocity and volume increased rapidly, while respiration slowed and declined.

The patient remained with me until 10.30 p.m., when he left very much improved and free from danger. A good night's rest completed the cure. The next day he reported that he took over 1 grain of hyoscyamin by the mouth, not by the syringe, and did not take any morphia. This was the correct statement, as he did not fully comprehend what he said when he first came to me. He had no desigh in misrepresenting the case, as he was fully aware that I knew of his opium proclivities, having been his medical attendant for five years, and having treated him in several severe illnesses. This case shows the power of a small quantity of morphia, used by the hypodermic method, to antagonise hyoscyamin without emesis.—*Medical and Surgical Reporter.*

